# Efficient Enzyme-Assisted Extraction and Conversion of Polydatin to Resveratrol From *Polygonum cuspidatum* Using Thermostable Cellulase and Immobilized β-Glucosidase

**DOI:** 10.3389/fmicb.2019.00445

**Published:** 2019-03-27

**Authors:** Chunqing Wang, Xiaolong Liu, Mengle Zhang, Haoyue Shao, Manman Zhang, Xiaomeng Wang, Qinghua Wang, Zhining Bao, Xinjiong Fan, He Li

**Affiliations:** ^1^School of Basic Courses, Guangdong Pharmaceutical University, Guangzhou, China; ^2^School of Basic Medical Sciences, Anhui Medical University, Hefei, China; ^3^Anhui Province Key Laboratory of Biomass Clean Energy, Department of Chemistry, University of Science and Technology of China, Hefei, China; ^4^Guangzhou Institute of Microbiology, Guangzhou, China

**Keywords:** resveratrol, *Polygonum cuspidatum*, cellulase, β-glucosidase, immobilization, deglycosylation

## Abstract

Resveratrol, a bioactive compound in high quantities in *Polygonum cuspidatum*, has well-known health benefits. However, it mainly exists in its glycosidic form, polydatin, in plants. To increase the production of resveratrol for various uses in medicine, foods, and cosmetics, an efficient deglycosylation technique is needed for converting polydatin into resveratrol. We screened a new cellulolytic strain of *Bacillus* from herb compost, and we optimized parameters within the fermentation process using response surface methodology with a Box–Behnken design. The yield of cellulase reached 2701.08 U/L, corresponding to values that were 5.4 times as high as those under unoptimized conditions. The *Bacillus* cellulase possessed good thermostablity and was stable under both acidic and neutral conditions. The cellulase was then used in the pretreatment of *P. cuspidatum* root. After incubation at 50°C for 4 h with shaking at 150 rpm, the contents of piceid and resveratrol were determined to be 7.60 ± 0.15 and 9.72 ± 0.29 mg/g, respectively. To obtain complete deglycosylation, immobilized β-glucosidase (bgl2238) was added to the cellulase-treated extracts of *P. cuspidatum* root to convert residual polydatin into resveratrol. After the first cycle, the contents of piceid and resveratrol were determined to be 0 and 13.69 ± 0.30 mg/g, respectively. Moreover, enzyme activity showed little loss during up to 4 consecutive cycles. These results demonstrated that the immobilized β-glucosidase possessed high deglycosylation activity and outstanding operational stability. The mixture of *Bacillus* cellulase and immobilized bgl2238 appears promising as a means to increase the supply of resveratrol in the medicine market worldwide.

## Introduction

Resveratrol (3,5,4′-trihydroxystilbene) is a natural polyphenolic compound widely used in medicines, foods, and cosmetic products (He et al., 2016). Recently, resveratrol has received increasing attention because it has beneficial properties for human health, including antimicrobial, antioxidant, anti-inflammatory, anti-aging, anticarcinogenic, and neuroprotective effects (Carter et al., 2014; He et al., 2016; Shen et al., 2017).

Resveratrol is widely found in a variety of food plants, including *Polygonum cuspidatum*, grapes, berries, and peanuts, among others (Nakata et al., 2012). However, plants contain only small concentrations of resveratrol (about 0.2% of dry weight), so obtaining large quantities of the compound is difficult and expensive. Resveratrol mainly exists as a glycoside, polydatin (3,5,4′-trihydroxystilbene-3-O-D-glucopyranoside), in plants (Chen et al., 2016). Polydatin has been reported to be present at concentrations that are 10 to 15 times higher than that of resveratrol (Wang et al., 2007; Chong et al., 2012). In addition, the content of polydatin is much higher in the dried root of *P*. *cuspidatum* (2% of dry weight) than in other sources (Kirino et al., 2012). Therefore, *P*. *cuspidatum* root potentially represents an excellent source for production of resveratrol (Wang et al., 2013). However, the bioavailability of polydatin is lower than that of resveratrol (Kuo et al., 2016), and studies have shown that human intestinal cells absorb polydatin with greater difficulty and more slowly than resveratrol (Signorelli and Ghidoni, 2005). Therefore, efficient extraction of polydatin from *P*. *cuspidatum* root followed by conversion of the compound to resveratrol represents a desirable goal.

Bioactive compounds from plant materials can be extracted through various techniques (Azmir et al., 2013), such as hot water extraction, microwave-based extraction, ultrasound-based extraction, and enzyme-based extraction (Wang et al., 2007). Compared with conventional methods, enzyme-based extraction facilitates greater extraction of major components from plant materials (Nagendra chari et al., 2013) and is an environmentally friendly process without toxic products (Kuo and Lee, 2009). Cellulase is a complex carbohydrase, including exo-1,4-β-glucanase (EC 3.2.1.91), endo-1,4-β-glucanase (EC 3.2.1.4), and β-glucosidase (EC 3.2.1.21) activities. It can effectively decompose cellulose through synergistic action of its component activities (Naika et al., 2007) to release intracellular polydatin and resveratrol. Afterward, β-glucosidase can transform polydatin into resveratrol by catalyzing the hydrolysis of β-glucosidic linkages. This cellulase-based extraction and conversion technology has been widely used in resveratrol production (Nagendra chari et al., 2013; Kuo et al., 2016; Mai et al., 2016).

However, the cost of enzymes is an important determinant in the economic feasibility of bioconversion, and the feasibility is affected by the enzymes’ high production costs, their lack of long-term stability, and difficulties in recycling and reusing them (Fan et al., 2017). Fully recyclable biocatalysts would help to minimize waste disposal and optimize economic benefits. In addition, enzyme immobilization might be the best method for improving operational stability, thus reducing the cost of production (Xie and Zang, 2017). The four classical immobilization methods include adsorption, entrapment, cross-linking, and covalent binding using commercial supports (Rodrigues et al., 2008). Among the various supports, chitosan is broadly applicable for immobilizing enzymes because it has desirable properties, such as hydrophilicity, biocompatibility, biodegradability, non-toxicity, and physiological inertness, and also possesses high affinity for proteins (Silva et al., 2012).

In the present work, a new cellulolytic strain of *Bacillus* from herb compost was screened based on the ability of the bacteria to grow on the sodium carboxymethylcellulose (CMC) medium. Response surface methodology (RSM) was used for optimizing various process parameters during the fermentation process. The bacterial cellulase was then used to pretreat *P. cuspidatum* root, and immobilized β-glucosidase (bgl2238) was added to the resultant extracts to convert residual polydatin into resveratrol. The biocatalysts were subsequently recycled and reused in additional rounds of extraction and conversion.

## Materials and Methods

### Chemicals and Materials

*Polygonum cuspidatum* root was gathered locally. Chemical reagents were of analytical or electrophoresis grade, and were purchased from Sigma-Aldrich (St. Louis, MO, United States). Molecular biological reagents were purchased from TaKaRa (Dalian, China) and used according to the instruction. Plasmid extraction kit and DNA extraction kit were purchased from OMEGA (Norcross, GA, United States).

### Isolation and Identification of Cellulolytic Strains of Bacteria

Cellulolytic strains of bacteria were isolated from herb compost by using serial dilutions and the pour plate technique. The isolated strain demonstrated a potential for cellulase production as shown by growth on plates of solid CMC stained with Congo red (Aulitto et al., 2017). Colonies were purified by repeated streaking. The purified colonies were further identified and screened to select cellulose-degrading strains. The liquid medium used to quantify the cellulase production level contained 1% CMC. Samples were collected every 24 h, centrifuged at 10,000 *g* for 5 min at 4°C, and used to measure extracellular cellulase activity at OD_540 nm_ (Pennacchio et al., 2018). One unit of enzyme activity was defined as the amount of enzyme catalyzing the release of 1 μmol of glucose equivalent per minute. Because of its remarkable enzymatic activity, strain no. 2 was selected for further study. Molecular identification was performed by 16S rRNA gene sequencing, and the 16S rRNA gene sequence was compared in the BLAST program provided by the NCBI and Nucleotide BLAST.

### Fermentation Experimental Design

Strain no. 2 clones were cultivated in a 250 mL flask containing 25 mL of CMC-Na liquid medium with shaking at 180 rpm at 28°C overnight. And then, the cells were transferred in fresh CMC-Na liquid medium with 2% inoculum size and incubated with shaking at 180 rpm at 28°C for 5 days. The supernatant was collected by centrifugation (8,000 *g* for 10 min at 4°C), and served as the crude enzyme for further study.

The Plackett–Burman experimental design was used to screen out and evaluate the relative importance of the medium’s different components as twelve runs experiment for CMCase production in submerged fermentation. Each variable was designated and used with a high (+) and low (−) concentration (Tables 1, 2). The nutrient factors tested included glucose, yeast extract, peptone, KH_2_PO_4_, inoculum size, medium volume, and initial pH.

**Table 1:** Factors and experimental domain for the screening with Placket–Burrman design.

Label	Parameter	Low level (−1)	High level (+1)
*A*	Glucose (g/L)	12	20
*B*	Yeast extract (g/L)	16	32
*C*	Peptone (g/L)	8	24
*D*	KH_2_PO_4_ (g/L)	4	12
*E*	Inoculum size (%)	6	14
*F*	Medium volume (mL)	40	60
*G*	Initial pH	5	7
*H*	–	−1	1
*J*	–	−1	1
*K*	–	−1	1
*L*	–	−1	1

**Table 2:** Plackett–Burman design for screening of parameters for cellulase production in fermentation.

Run No.	*A*	*B*	*C*	*D*	*E*	*F*	*G*	*H*	*J*	*K*	*L*	Relative activity (%)
1	1	−1	−1	−1	1	−1	1	1	−1	1	1	88.5
2	−1	1	1	1	−1	−1	−1	1	−1	1	1	63.37
3	−1	−1	−1	−1	−1	−1	−1	−1	−1	−1	−1	61.55
4	1	−1	1	1	−1	1	1	1	−1	−1	−1	100
5	1	1	−1	1	1	1	−1	−1	−1	1	−1	53.78
6	−1	1	1	−1	1	1	1	−1	−1	−1	1	86.28
7	1	−1	1	1	1	−1	−1	−1	1	−1	1	61.76
8	−1	−1	−1	1	−1	1	1	−1	1	1	1	80.93
9	−1	1	−1	1	−1	−1	1	1	1	−1	−1	84.56
10	−1	−1	1	−1	1	1	−1	1	1	1	−1	71.85
11	1	1	−1	−1	1	1	−1	1	1	−1	1	65.19
12	1	1	1	−1	−1	−1	1	−1	1	1	−1	74.07

In order to optimize process conditions, a Box–Behnken design (BBD) was used for evaluating, as dependent variable, cellulase production. The independent variables were concentrations of yeast extract, medium volume, and pH, and the levels are detailed in Tables 3, 4.

**Table 3:** ANOVA results of quadratic model for cellulase production (*R*^2^ = 0.9997).

Parameter	Sum of squares	Degrees of freedom	Mean square	*F* value	*P* value
*A*	2.29	1	2.29	567.31	0.0267
*B*	116.19	1	116.19	28807.35	0.0038
*C*	43.40	1	43.40	10759.35	0.0061
*D*	0.77	1	0.77	190.94	0.0460
*E*	0.22	1	0.22	54.22	0.0859
*F*	48.88	1	48.88	12120.01	0.0058
*G*	1560.43	1	1560.43	3.869 × 10^5^	0.0010
*H*	253.00	1	253.00	62727.48	0.0025
*J*	19.05	1	19.05	4723.44	0.0093
*K*	60.03	1	60.03	14884.00	0.0052
Residual	4.033 × 10^−3^	1	4.033 × 10^−3^		
Cor. Total	2104.27	11			

**Table 4:** Experimental ranges and levels with Box–Behnken design.

Factors	Range and Level		
	−1	0	1
*A*: Yeast extract (g/L)	15	20	25
*B*: Medium volume (mL)	40	50	60
*C*: initial pH	5.5	6.5	7.5

### Effect of Temperature and pH on Activity and Stability of *Bacillus* Cellulase

After centrifugation (8,000 *g* for 10 min at 4°C) of fermentation broth, the supernatant was collected and served as the crude enzyme for further study. The optimal temperature was determined by testing *Bacillus* cellulase activity at pH 5.0 within a temperature range of 5 to 80°C. The cellulase activity was measured as previously described (Pennacchio et al., 2018), with slight modifications. Thermostability was determined by pre-incubation of the cellulase in 50 mM citric acid-NaOH buffer (pH 5.0) at various temperatures from 5 to 80°C for 4 h. Then, the residual was evaluated. The optimal pH of cellulase was next tested at 50°C. The buffers (with the final concentration of 50 mM) were as follows: glycine-HCl buffer (pH 2.0 to 3.6), citric acid-NaOH buffer (pH 3.5 to 5.5), potassium phosphate buffer (pH 5.0 to 7.0), Tris–HCl buffer (pH 6.5 to 9.0), and glycine-NaOH buffer (pH 8.5 to 10.0). Overlapping pH values were used to validate that there were no buffer effects on substrate decomposing. The pH stability was tested after incubation of *Bacillus* cellulase for 4 h at 50°C in the previous solution.

### Cellulase-Assisted Extraction and Conversion of Polydatin of *P. cuspidatum* Root Into Resveratrol

Dried *P. cuspidatum* root was ground to powder and passed through a 100- to 150-mesh sieve. The grain size of the resulting powder was about 0.10 to 0.15 mm. One gram of the powder was extracted with 10 mL of citric acid-NaOH buffer (50 mM, pH 5.0) containing 27.0 U cellulase. After incubation at 50°C for 4 h with shaking at 150 rpm, the solution (10 mL) was extracted with methanol (40 mL) at normal temperature for 2 h with shaking at 150 rpm. The amounts of polydatin and resveratrol were monitored spectrophotometrically by Labsystems Dragon Wellscan MK3 at 325 and 306 nm, respectively.

### Immobilization of β-Glucosidase Onto Glutaraldehyde-Activated Chitosan Beads

We identified a novel β-glucosidase gene, *bgl2238* (GenBank: KU320675.1), from a soil metagenomic library by functional screening (Tang et al., 2018). The full length of the gene was 2 238 bp, which encoded a protein of 745 amino acids. The β-glucosidase had a molecular mass of 80.67 kDa with an isoelectric point (pI) of 4.95. The maximal activity (29.1 U/mg) of the enzyme was observed at 44°C and pH 6.0 with 4-nitrophenyl-β-D-glucopyranoside (pNPG) as the substrate; furthermore it retained 70% of its activity within the temperature range of 4–50°C for 4 h. The *K*_m_ and *V*_max_ values of bgl2238 were 0.296 mmol/L and 576 μmoL/(L⋅min), respectively.

To enhance the stability and to recycle biocatalysts, β-glucosidase (bgl2238) was immobilized on chitosan beads using glutaraldehyde as the crosslinker (Liburdi et al., 2016), with some modifications. Chitosan solution (2% w/v) was added into a solution of glacial acetic acid (1% v/v) and stirred magnetically at 37°C for 1 h. The mixture was stored at 4°C until air bubbles were gone. Then, the chitosan solution was added dropwise into 1 M NaOH solution containing 30% methanol, using a 1-mL syringe. After hardening, chitosan beads were treated with 100 mL of glutaraldehyde solution (0.1%) in sodium phosphate buffer (pH 6.0) for activation. The process was carried out at room temperature under shaking at 150 rpm for 3 h. The activated beads were then continuously washed with distilled water to remove unreacted glutaraldehyde. One hundred milligrams of activated beads was dispersed in 1 mL of bgl2238 (96 U/mL, 0.32 mg/mL), which was immobilized at room temperature under shaking at 200 rpm for 1 h. Finally, the beads were washed to remove unbound enzyme and then stored at 4°C until further study.

The immobilization parameters (glutaraldehyde concentration, activation time and temperature, and coupling time) were investigated. Different glutaraldehyde concentrations (0 to 3%) were used to optimize the immobilization. In addition, to analyze the effect of activation time, beads were activated with 0.5% glutaraldehyde at 20°C for different time intervals (0.5 to 3 h). Beads were activated with 0.5% glutaraldehyde for 1 h at different temperature (5 to 50°C) to analyze the effect of activation temperature. Finally, to determine the effect of coupling time, the enzyme was cross-linked to the activated beads for different time intervals (1 to 6 h). All experiments were done in triplicate.

The activity of the immobilized enzyme was measured using 4-nitrophenyl β-D-glucuronide (pNPG) as a substrate (Mai et al., 2013). Fifty milligrams of immobilized enzyme was mixed with 200 μL of 50 mM pNPG in 50 mM potassium phosphate buffer (pH 6.0) and incubated at 44°C for 20 min. The reaction was then terminated by adding 400 μL of 1 M Na_2_CO_3_. The concentration of pNP was determined by measuring the absorbance of the solution at 405 nm (Cao et al., 2015). One unit of enzymatic activity was defined as the amount of β-glucosidase that releases 1 μmol pNP from pNPG per minute under reaction conditions. The immobilized β-glucosidase was dried at normal temperature and then stored at 4°C. The activity recovery (%) = (Immobilized enzyme activity/Total enzyme activity) × 100%.

### Comparison of Catalytic Properties of Free and Immobilized Enzyme

The effects of pH and temperature on the activity of free and immobilized β-glucosidase were determined by using pNPG as a substrate. The optimal pH was tested at pH 6. The pH buffers included citric acid-NaOH buffer (pH 3.0 to 5.5), potassium phosphate buffer (pH 5.0 to 7.0), and Tris–HCl buffer (pH 6.5 to 9.0). The pH stability was tested after incubation of the purified enzyme for 24 h at 30°C in the different buffers. The optimal temperature was determined by testing free and immobilized enzyme activity at pH 6.0 within a temperature range of 35 to 60°C. The activity of the immobilized enzyme was measured using 4-nitrophenyl β-D-glucuronide (pNPG) as a substrate. Thermostability was evaluated by pre-incubation of the cellulase in 50 mM citric acid-NaOH buffer (pH 6.0) at various temperatures from 35 to 60°C for 4 h. The optimal temperature and thermostability were determined according to the method above. Thermostability was measured by preincubation of the purified enzyme in 50 mM potassium phosphate buffer (pH 6.0) at 35 to 60°C for 4 h.

### Conversion of Polydatin Into Resveratrol by Immobilized β-Glucosidase in the Cellulase-Assisted Extracts of *P. cuspidatum* Root

One gram of the root powder was extracted with 10 mL of citric acid-NaOH buffer (50 mM, pH 5.0) containing 27.0 U of cellulase at 50°C for 6 h with shaking at 150 rpm. After that, the mixture was adjusted to pH 6.0, and then 0.1 g of immobilized enzyme was added to the reaction mixtures. After incubation at 50°C for 1 h with shaking at 150 rpm, the suspension was filtered to recover immobilized enzymes. To determine the amount of resveratrol produced in the first reaction, the reaction mixtures underwent high performance liquid chromatography (HPLC) analysis according to a previously reported method (Mai et al., 2016). The immobilized enzymes were repeatedly used to catalyze the conversion for up to 10 batch reactions to determine their operational stability. The activity of the immobilized β-glucosidase was tested using pNPG as a substrate. The residual activity (%) was determined as follows:

$$\mathrm{Residual activity}\;(\%)=\frac{\mathrm{enzyme activity in}\;n\mathrm{th cycle}}{\mathrm{enzyme activity in 1st cycle}}\times 100\%$$

## Results and Discussion

### Isolation and Identification of Cellulolytic Strains

Hundreds of colonies from CMC plates were tested for the ability to grow in liquid medium. Based on extracellular cellulase activity, strain no. 2 had the highest optical absorbance at 540 nm. The 16S rRNA sequence was listed in Table 5. Sequencing of the 16S rRNA gene of this strain showed that its nearly full-length gene sequence (>1400 bp) had 99% identity with *Bacillus subtilis* according to a BLAST query.

**Table 5:** The 16S rRNA sequence of stain no. 2.

GCTATAATGCAAGTCGAGCGGACAGATGGGAGCTTGCTCCCTGATGTTAGCGGCGGACGGGTGAGTAACACGTGGGTAACCTGCCTGTAAGACTGGGATAACTCCGGGA AACCGGGGCTAATACCGGATGGTTGTTTGAACCGCATGGTTCAAACATAAAAGGTGGCTTCGGCTACCACTTACAGATGGACCCGCGGCGCATTAGCTAGTTGGTGAGGT AACGGCTCACCAAGGCAACGATGCGTAGCCGACCTGAGAGGGTGATCGGCCACACTGGGACTGAGACACGGCCCAGACTCCTACGGGAGGCAGCAGTAGGGAATCTT CCGCAATGGACGAAAGTCTGACGGAGCAACGCCGCGTGAGTGATGAAGGTTTTCGGATCGTAAAGCTCTGTTGTTAGGGAAGAACAAGTACCGTTCGAATAGGGCGGTA CCTTGACGGTACCTAACCAGAAAGCCACGGCTAACTACGTGCCAGCAGCCGCGGTAATACGTAGGTGGCAAGCGTTGTCCGGAATTATTGGGCGTAAAGGGCTCGCAGG CGGTTTCTTAAGTCTGATGTGAAAGCCCCCGGCTCAACCGGGGAGGGTCATTGGAAACTGGGGAACTTGAGTGCAGAAGAGGAGAGTGGAATTCCACGTGTAGCGGTGA AATGCGTAGAGATGTGGAGGAACACCAGTGGCGAAGGCGACTCTCTGGTCTGTAACTGACGCTGAGGAGCGAAAGCGTGGGGAGCGAACAGGATTAGATACCCTGGTAG TCCACGCCGTAAACGATGAGTGCTAAGTGTTAGGGGGTTTCCGCCCCTTAGTGCTGCAGCTAACGCATTAAGCACTCCGCCTGGGGAGTACGGTCGCAAGACTGAAACT CAAAGGAATTGACGGGGGCCCGCACAAGCGGTGGAGCATGTGGTTTAATTCGAAGCAACGCGAAGAACCTTACCAGGTCTTGACATCCTCTGACAATCCTAGAGATAGG ACGTCCCCTTCGGGGGCAGAGTGACAGGTGGTGCATGGTTGTCGTCAGCTCGTGTCGTGAGATGTTGGGTTAAGTCCCGCAACGAGCGCAACCCTTGATCTTAGTTGCC AGCATTCAGTTGGGCACTCTAAGGTGACTGCCGGTGACAAACCGGAGGAAGGTGGGGATGACGTCAAATCATCATGCCCCTTATGACCTGGGCTACACACGTGCTACAA TGGACAGAACAAAGGGCAGCGAAACCGCGAGGTTAAGCCAATCCCACAAATCTGTTCTCAGTTCGGATCGCAGTCTGCAACTCGACTGCGTGAAGCTGGAATCGCTAGT AATCGCGGATCAGCATGCCGCGGTGAATACGTTCCCGGGCCTTGTACACACCGCCCGTCACACCACGAGAGTTTGTAACACCCGAAGTCGGTGAGGTAACCTTTTAGGA GCCAGCCGCCGAAG

### Fermentation Experimental Design for Cellulase Production

The isolated no. 2 strain had potential cellulase production as determined by its growth on CMC plates and in liquid medium. RSM is suitable for fermentation optimization (Tabssum et al., 2018). Compared with one-factor-at-a-time experiments, statistically designed experiments can reveal the effects of interactions among the factors in linear and quadratic terms.

Various process parameters were optimized and nutritional conditions were screened using the Plackett–Burman design of RSM. Table 1 shows the main nutritional components and their levels. The amount of cellulase was chosen as the observed response to determine the effects of the variables studied. Twelve run experiments were performed for screening of various nutrients for cellulase production, and the results are presented in Table 2. The final regression equation in terms of coded factors was determined by Design-expert software, which yielded Eq. (1):

(1) $$\begin{array}{c} R=+8.87139-0.21833\text{A}-1.55583\text{B}+0.95083\text{C}\\ \;-0.063333\text{D}+0.033750\text{E}+0.26911\text{F}+11.40333\text{G}\\ \;+4.59167\text{H}-1.26000\text{J}-2.23667\text{K} \end{array}$$

In Eq. (1), *R* is the dependent variable (the predicted response of cellulase yield); *A* to *K* are the independent variables. The statistical significance of Eq. (1) was checked by the *F*-test, and the analysis of variance (ANOVA) for response surface quadratic model is summarized in Table 3. Results showed that 3 variables (i.e., yeast extract, medium volume, and initial pH) were significant for cellulase production in submerged fermentation using *B. subtilis*. Yeast extract had a significant effect on cellulase production in submerged fermentation using *B. subtilis* (Li et al., 2008). A previous study also revealed yeast extract as the best nitrogen source for cellulase production by *B. cereus* MRLB1 (Afzal et al., 2012).

To optimize the yeast extract concentrations, medium volume, and initial pH, the Box–Behnken design of RSM with 3 levels was used, and the results are presented in Tables 4, 6. The response obtained was calculated with a second-order polynomial regression equation, which yielded Eq. (2). In Eq. (2), *R* is the predicted response of cellulase yield; *A*, *B*, and *C* are the coded values of the test variables, yeast extract, medium volume, and initial pH, respectively. The statistical significance of Eq. (2) was checked by the *F*-test, and ANOVA for the response surface quadratic model is summarized in Table 7.

**Table 6:** Box–Bhenken design for cellulase production.

Run	*A*	*B*	*C*	Relative activity (%)	Activity (U/L)
1	0	0	0	95.04	1667.98
2	1	0	−1	78.62	1379.80
3	−1	0	1	72.37	1270.11
4	1	−1	0	80.27	1408.76
5	0	1	−1	79.07	1387.70
6	1	1	0	82.85	1454.04
7	0	−1	−1	63.73	1118.48
8	−1	0	−1	62.68	1100.05
9	−1	1	0	88.04	1545.13
10	1	0	1	87.86	1541.97
11	0	−1	1	81.19	1424.91
12	0	0	0	97.77	1715.89
13	0	0	0	100	1755.03
14	0	1	1	85.67	1503.53
15	0	0	0	99.38	1744.15
16	−1	−1	0	67.47	1184.12
17	0	0	0	96.29	1689.92

**Table 7:** ANOVA results of quadratic model for cellulase production (*R*^2^ = 0.9602).

Parameter	Sum of squares	Degrees of freedom	Mean square	*F* value	*P* value
*A*	190.52	1	190.52	14.36	0.0068
*B*	230.48	1	230.48	17.38	0.0042
*C*	231.34	1	231.34	17.44	0.0042
AB	80.91	1	80.91	6.10	0.0428
AC	0.051	1	0.051	3.817 × 10^−3^	0.9525
BC	29.32	1	29.32	2.21	0.1806
A^2^	423.73	1	423.73	31.95	0.0008
B^2^	269.93	1	269.93	20.35	0.0028
C^2^	635.12	1	635.12	47.88	0.0002
Residual	92.84	7	12.26		
Cor. Total	2334.11	16			

(2) $$\begin{array}{c} R=+97.696+4.488\text{A}+5.3675\text{B}+5.37750\text{C}\\ \;-4.4975\text{AB}-0.11250\text{AC}-2.7075\text{BC}-10.03175{\text{A}}^{2}\\ \;-8.00675{\text{B}}^{2}-12.28175{\text{C}}^{2} \end{array}$$

**FIGURE 1:**
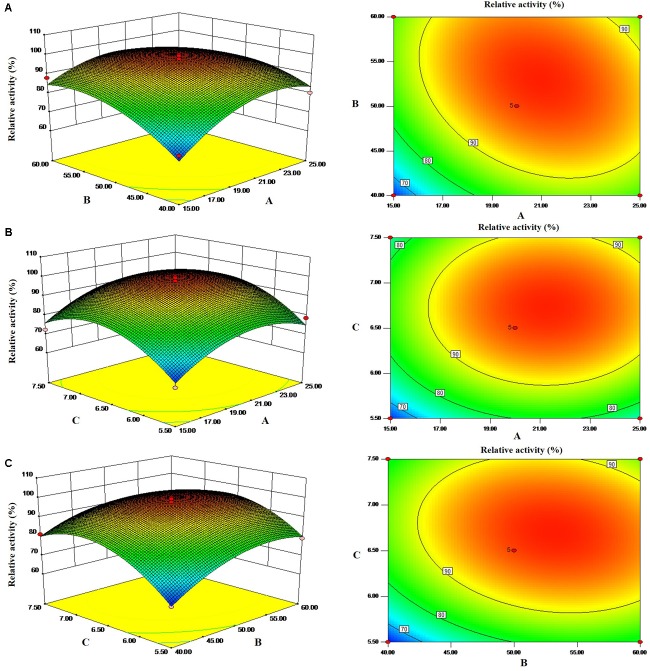
Response surfaces and corresponding contours of cellulase production in terms of interaction between two parameters. **(A)** Yeast extract (g/L) and medium volume (mL). **(B)** Yeast extract (g/L) and initial pH. **(C)** Medium volume (mL) and initial pH.

The 3-dimensional response surface plots generated from the Box–Behnken design experiments are the graphical representations of the regression equation, and these plots are shown in Figure 1. The main goal of RSM is to efficiently track the optimal values of the process parameters such that the response is maximized. Software-based numerical optimization of the overall desirability function was carried out to find the optimal response within the specified ranges of the variables in the optimization of fermentation for cellulase production. The results showed that maximum cellulase production occurred with a concentration of 20 g/L yeast extract, 50 mL medium volume, and initial pH 6.5. Previous studies reported that initial medium pH of 7.0 to 7.2 was the most favorable for cellulase production by *Bacillus* sp. in submerged fermentation (Rastogi et al., 2009; Bai et al., 2012). In another study, an initial medium pH of 9.0 was optimized through RSM for cellulase production by *Bacillus cereus* (Tabssum et al., 2018).

The optimal experimental conditions were as follows: 20 g/L yeast extract, 50 mL medium volume, initial pH 6.5, 14 g/L glucose, 22 g/L peptone, 6 g/L KH_2_PO_4_, and 10% inoculum size with shaking at 28°C for 48 h. The predicted yield of cellulase was 2743.29 U/L. Under these optimal experimental conditions, the yield of cellulase by the no. 2 strain reached 2701.08 U/L. The predicted cellulase production was close to the observed value, highlighting the accuracy of the model. The yield of cellulase was corresponding to a value that was 5.4 times as high as those under unoptimized conditions, which was much higher than cellulase activity of *Penicillium* sp. LMI01 (Santa-Rosa et al., 2018). Successful production is a very attractive property for enzymes used in practical applications.

### Effect of Temperature and pH on Activity and Stability of *Bacillus* Cellulase

**FIGURE 2:**
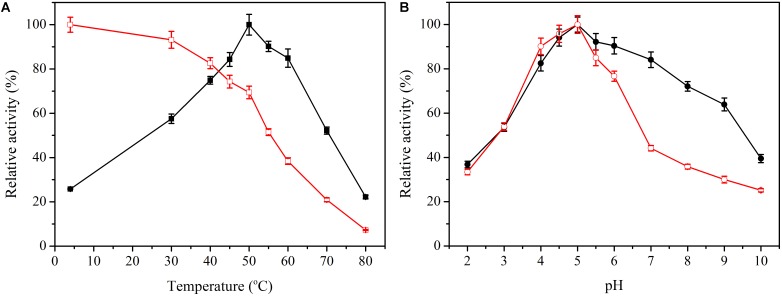
Effect of temperature and pH on activity and stability of *Bacillus* cellulase. **(A)** Effect of temperature on activity (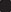) and stability (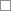) of *Bacillus* cellulase. **(B)** Effect of pH on activity (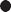) and stability (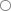) of *Bacillus* cellulase. Data points are the average of triplicate measurements, and error bars represent the standard deviation.

To measure the optimal temperature of *Bacillus* cellulase, the catalytic activity was assessed at different temperatures between 5 and 80°C with CMC as a substrate. *Bacillus* cellulase showed the highest activity at 50°C (Figure 2A), and it retained 20% of the maximum activity at 80°C. Moreover, it maintained more than 50% of its maximal activity at 30 to 70°C, indicating that *Bacillus* cellulase possesses good adaptability to moderate and high temperatures. The optimal temperature of this cellulase was the same as CMCase from *Bacillus* sp. (Vijayaraghavan and Vincent, 2012). Thermostability was determined by analysis of the residual activity toward CMC after preincubation for 4 h at temperatures ranging from 4 to 80°C. This cellulase exhibited excellent thermostability (Figure 2A). After preincubation at 60°C for 4 h, it retained as much as 38.4% of its total activity, similar to other cellulase from *Bacillus* sp. (Yin et al., 2010; Vijayaraghavan and Vincent, 2012), which proved that it possessed good thermostablity.

The optimal pH of this cellulase was 5.0, which differed from other cellulases. The pH 6.0 was optimal in *Bacillus pumilus* EB3 (Ariffin et al., 2006), and pH 7.0 was optimal in *B. subtilis* YJI (Yin et al., 2010). CMCase was most active at pH 8.0 in *Bacillus amyloliquefaciens* DL-3 (Lee et al., 2008). Interestingly, the cellulase exhibited excellent activity over a wide pH range from 3.0 to 9.0 (Figure 2B), retaining 50% relative activity. Good pH adaptability is indispensable because conditions are frequently changeable during enzyme use. With regard to pH stability, this cellulase retained more than 50% activity in the pH range 3.0 to 6.0, but the activity abruptly declined between 6 and 7 (Figure 2B). Thus, the pH has an obvious influence on the cellulase activity, which is stable in both acidic and neutral conditions.

### Cellulase-Assisted Extraction and Conversion of Polydatin Into Resveratrol of *P. cuspidatum* Root

The optimal conditions were tested with extracts of *P. cuspidatum* root, which contained high amounts of polydatin and resveratrol. Enzymatic extraction of polydatin and resveratrol was determined with spectrophotometric methods. The contents of piceid and resveratrol were determined to be 7.60 ± 0.15 and 9.72 ± 0.29 mg/g, respectively. The yield of resveratrol was obtained through cellulase-based extraction, and it was slightly higher than resveratrol production by piceid deglycosylation using cellulase (Kuo et al., 2016), but lower than the highest yield (13.5 mg/g) of resveratrol through fermentation by *Aspergillus oryzae* (Wang et al., 2007). Although the total yield of polydatin and resveratrol was satisfactory, much of the polydatin in the extracts was not completely converted into resveratrol by cellulase under the optimal conditions. The results demonstrated that the *Bacillus* cellulase effectively broke down cellulose through synergistic action to release polydatin and resveratrol contained inside *P. cuspidatum* root, but its β-glucosidase had low deglycosylation activity.

### Immobilization of β-Glucosidase Onto Glutaraldehyde-Activated Chitosan Beads

Successful application of a catalyst depends to a significant extent on its stability and recyclability, and immobilization of enzymes onto suitable materials is a proven strategy. In the current study, chitosan and glutaraldehyde were employed. β-glucosidase was covalently immobilized onto glutaraldehyde-activated chitosan beads, and the following immobilization parameters were investigated: glutaraldehyde concentration, activation time and temperature, and coupling time.

**FIGURE 3:**
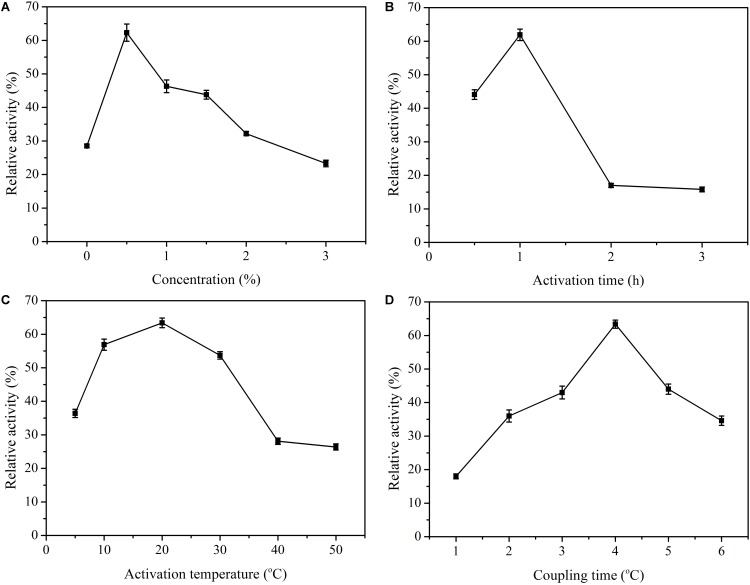
Immobilization conditions of β-glucosidase onto glutaraldehyde-activated chitosan beads. **(A)** The relative activity with glutaraldehyde concentration. **(B)** The relative activity for activation time. **(C)** The relative activity at activation temperature. **(D)** The relative activity for coupling time. Data points are the average of triplicate measurements, and error bars represent the standard deviation.

As the concentration of glutaraldehyde increased, the loading efficiency of enzymes also increased because of the presence of a large number of aldehyde groups for binding the enzyme molecules. Nevertheless, when the relative activities of enzymes were examined, higher enzyme activities were generally observed for lower glutaraldehyde concentrations. Its optimal value was 0.5% (Figure 3A), and further increase of the concentration did not increase the amount of bound enzyme. The possible explanation for a sharp decrease in this case could be the formation of more covalent bonds, which facilitated structural rigidity of the β-glucosidase, resulting in decreased enzyme activity. The obtained glutaraldehyde concentration was different from other studies in the literature. Altun and Cetinus (2007) found that 1% concentration was the optimum for pepsin immobilization. In another study in which lignin peroxidase and manganese peroxidase enzymes were co-immobilized with chitosan microspheres, the highest enzyme activity for both enzymes was obtained with 0.75% glutaraldehyde-activated spheres (Ran et al., 2011). Monsan (1978) proved that the amounts of both the support-bound glutaraldehyde and the immobilized enzyme increased with an increase in the glutaraldehyde concentration from 0.1 to 5%. A glutaraldehyde concentration of 4.74% was shown to be the optimum for β-D-galactosidase using PEI activated CP beads (Wahba, 2017). Different enzyme concentrations may explain this result. Aldehyde groups are formed on the surface of chitosan with the use of glutaraldehyde at the appropriate concentration and after activation is performed for the required time. Different activation times were tried for optimal immobilization, and at 1 h, the relative activity was maximal (Figure 3B). After 1 h, little enzyme activity was obtained. These results indicated that an excessive number of aldehyde groups on the carrier may lead to an excessive cross-linking reaction, which changes the conformation of the enzymes and decreases the relative activities. Afterward, activation temperatures were also investigated. The results clearly showed that the activation temperature played an insignificant role in the immobilization process (Figure 3C). The recovery of enzyme activity was more than 50% at temperatures ranging from 10 to 30°C. Finally, for the coupling time, the optimal value was 4 h (Figure 3D). Therefore, the overall optimal conditions were a glutaraldehyde concentration of 0.5%, an activation time of 1 h, an activation temperature of 20°C, and a coupling time of 4 h. Under such conditions, the activity recovery remained at 68.3%.

### Comparison of Catalytic Properties of Free and Immobilized Enzyme

**FIGURE 4:**
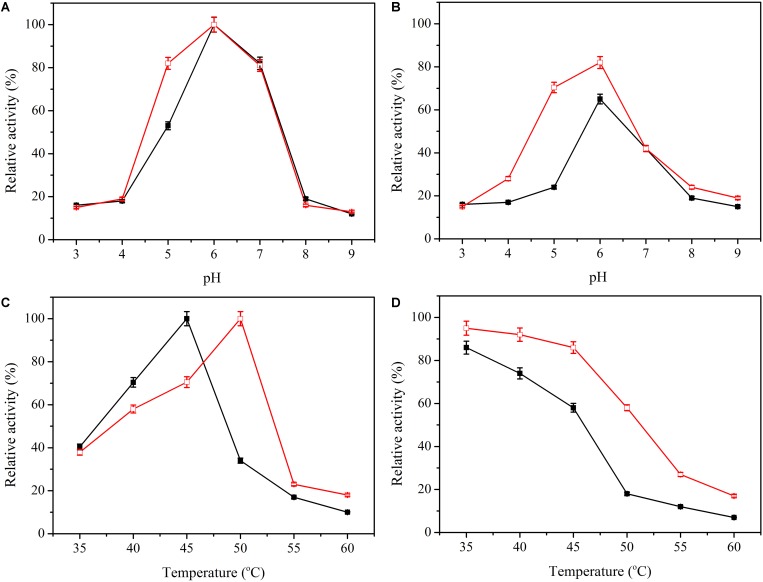
Effect of pH and temperature on the activity and stability of free (black line) and immobilized β-glucosidase (red line). **(A)** Effect of pH on activity of free (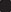) and immobilized β-glucosidase (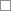). **(B)** Effect of pH on stability of free (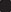) and immobilized β-glucosidase (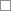). **(C)** Effect of temperature on activity of free (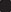) and immobilized β-glucosidase (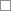). **(D)** Effect of temperature on stability of free (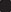) and immobilized β-glucosidase (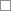). Data points are the average of triplicate measurements, and error bars represent the standard deviation.

Activity of enzyme can be significantly affected by pH and temperature. Here, the influence of pH on activity of enzyme was tested between pH 3.0 and pH 9.0. The optimal pH of the immobilized β-glucosidase was 6.0, the same as the soluble enzyme (Figure 4A). However, the relative activity at pH 4 to 6 was higher than the soluble enzyme. Excellent pH adaptability is attractive when dealing with complicated conditions present during use of an enzyme. The pH adaptability was altered (Figure 4B), and the immobilized enzyme maintained better adaptability over a broad range of pH values.

The activity of the soluble and immobilized enzyme was determined at temperatures of 35 to 60°C. The immobilized β-glucosidase had a higher optimal temperature of 50°C, 6 degrees higher than that of the soluble enzyme (Figure 4C). Thermostability of β-glucosidase was determined by testing the residual activity after preincubation at 35 to 60°C for 4 h (Figure 4D). The immobilized enzyme exhibited excellent thermostability. After preincubation at 50°C for 4 h, the immobilized enzyme retained 58.4% of its total activity, whereas the soluble enzyme was nearly deactivated. For practical applications, thermostability enhancement is generally attractive because it extends the service life of the enzyme in reaction (Fan et al., 2017).

### Conversion of Polydatin Into Resveratrol by Immobilized β-Glucosidase in the Cellulase-Assisted Extracts of *P. cuspidatum* Root

**FIGURE 5:**
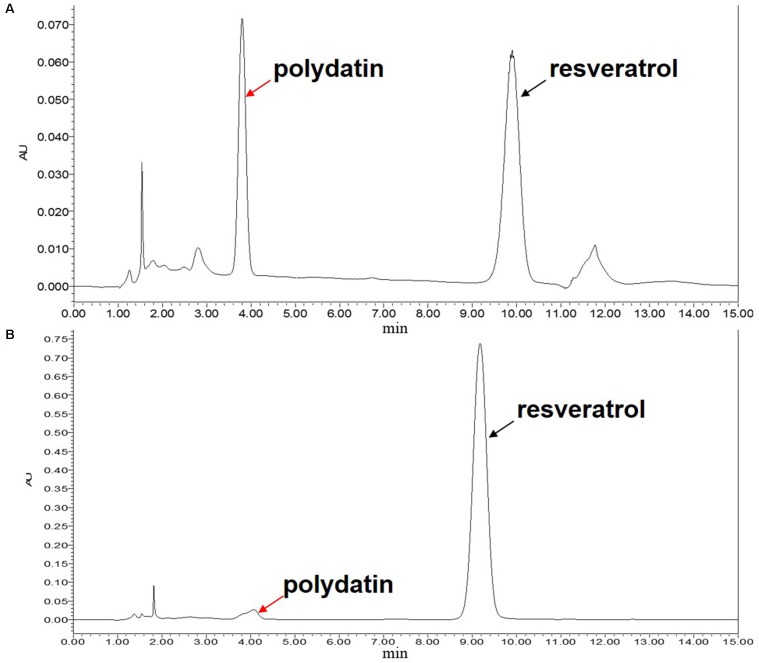
HPLC analysis of polydatin and resveratrol before **(A)** and after **(B)** conversion of immobilized β-glucosidase after the first cycle. The black and red arrows denote polydatin and resveratrol peaks, respectively.

In the above experiment, the total yield of polydatin and resveratrol extracted by *Bacillus* cellulase was satisfactory, but the extracts still had a considerable amount of polydatin. To obtain complete deglycosylation, immobilized β-glucosidase (bgl2238) was added to the cellulase-digested extracts of *P. cuspidatum* root to convert residual polydatin into resveratrol. The ability to convert polydatin into resveratrol was determined by spectrophotometric methods and HPLC analysis. The results are shown in Figure 5. The immobilized enzyme was able to efficiently convert polydatin. In the first cycle, the contents of piceid and resveratrol were 0 and 13.69 ± 0.30 mg/g, respectively. The resveratrol yield was much higher than resveratrol production through piceid deglycosylation using cellulase (Kuo et al., 2016) and was similar to the highest yield (13.5 mg/g) of resveratrol fermented by *A. oryzae* (Wang et al., 2007).

**FIGURE 6:**
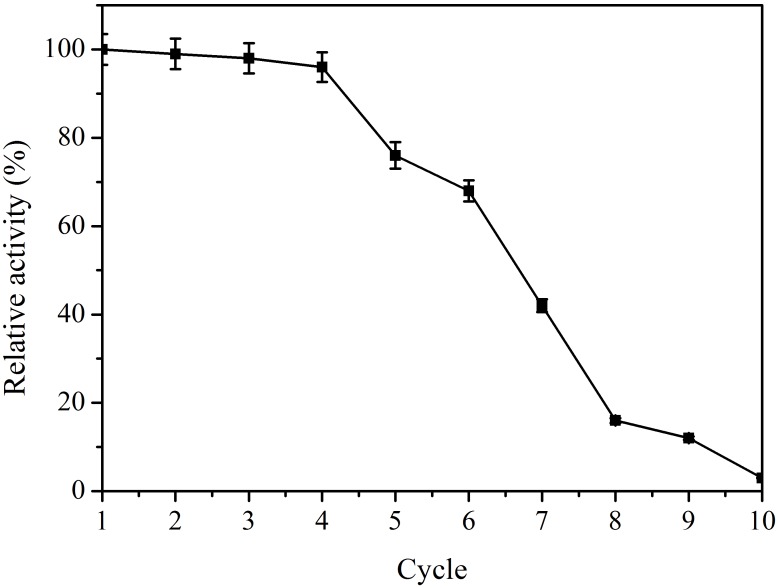
Operational stability of the immobilized β-glucosidase. The immobilized enzyme was incubated with the cellulase-digested extracts of *P. cuspidatum* root at 50°C for 6 h with shaking at 150 rpm, and then repeatedly used for up to 10 batch reactions.

The operational stability of immobilized enzymes is very important for industrial applications. The operational stability of the immobilized bgl2238 on glutaraldehyde-activated chitosan beads was investigated for 10 consecutive cycles (Figure 6). Little loss in enzyme activity occurred up to 4 consecutive cycles. After 4 cycles, a sharp decrease was observed, which may have been due to heat inactivation. These results suggest that the immobilized β-glucosidase possesses high deglycosylation activity and outstanding operational stability, which may be useful in the conversion of polydatin to resveratrol.

## Conclusion

Recently, resveratrol has received increasing attention because it may have beneficial effects on human health through its physiological activities. However, resveratrol mainly exists in plants in its glycosidic form, polydatin. To increase the production of resveratrol, *Bacillus* cellulases were produced and used to break down cellulose through synergistic action to release intracellular polydatin and resveratrol from *P. cuspidatum* root. However, the β-glucosidase in the plant tissue possessed low deglycosylation activity. To obtain complete deglycosylation and to permit recycling of biocatalysts, immobilized β-glucosidase (bgl2238) was added to the cellulase-digested extracts of *P. cuspidatum* root to convert residual polydatin into resveratrol. The mixture (*Bacillus* cellulase and immobilized bgl2238) provided a high extraction yield and efficient deglycosylation activity, and it may offer a promising technique to increase the supply of resveratrol in the medicine market worldwide.

## Author Contributions

CW have screened a new cellulolytic strain and optimized its optimal conditions. XL have immobilized of β-glucosidase onto chitosan beads. MeZ have extracted and polydatin and resveratrol of *P. cuspidatum* root using *Bacillus cellulases*. HS have done HPLC analysis. MaZ have determined operational stability of the immobilized enzyme. XW have tested deglycosylation activity of immoblized β-glucosidase. QW have tested the activity of *Bacillus* cellulases. ZB have tested the activity of immoblized β-glucosidase. XF have written and revised the manuscript. HL have conceived the study, supervised the experiments. All authors have read and approved the manuscript.

## Conflict of Interest Statement

The authors declare that the research was conducted in the absence of any commercial or financial relationships that could be construed as a potential conflict of interest.
